# The Nursing Supplement

**Published:** 1890-01-04

**Authors:** 


Being the Extra Nursing Supplement of "The Hospital" Newspaper.

All Contributions for this Supplement should be addressed to the Editor, The Hospital, 139-140, Salisbury Court, Fleet Street, London, E.C., and should have the word "Nursing" plainly written in left-hand top corner of the envelope.

### En Passant.

DOLL NURSES.—Many are the applications now coming in from hospitals anxious to hold an exhibition of nursing uniforms, and our dolls are making engagements for their provincial tour. On January 13, 14, and 15 they will be on view at the Hahnemann Hospital, Liverpool. Further particulars as to hours, etc., will be given in our next number. If any Northern hospitals not represented in London would like to join the exhibition now would they please send their dolls to the matron of the above hospital. We hope all nurses in Liverpool and Manchester will manage to find time to see the exhibition while it is on view at the Hahnemann Hospital.

CHURCH ARMY NURSES.—We are requested by the authorities of the Church Army to say that they do not profess to send forth "trained" nurses. On their printed papers they state them to be "working" nurses. We are informed that they have some slight training, however, which appears, on the whole, to give a very considerable satisfaction to the clergy under whom they work; so much so, that it is quite impossible for the society to provide anything like the number of their "working" nurses. Would it not be as well to call these women "evangelist nurses," or some such term, so that the public might not be mistaken as to the fact of the training?

NURSES FOR INDIA.—Not long ago the India Office was obliged to refuse to send more nurses to India, and at once Lady Roberts supplied from her fund eight highly-trained ladies, stationing them where they were most urgently needed—namely, two each at Meerut, Miankin, Pareilly, and Lucknow. Reports speak of the alarming increase of late years in the mortality from enteric fever among the troops, resulting in 266 deaths during the year past in the Bombay Presidency alone. At Chakrata, a hill depot for troops, the percentage of deaths was fifty-two, but here were no nurses, while at Meerut, a similar establishment, only supplied with nurses, it was only 17 per cent. In all cases, however—the plains as well as the hills—an equally satisfactory diminution has been observed when placed under the care of these ladies.

NORTHAMPTON INSTITUTE.—Miss Henrietta Stewart, now of Northampton, owes her training, in 1872, to St. John's House in the wards of King's College Hospital, where she became "sister" of medical and surgical wards for several years; then occupied the same post at the County Hospital, Leicester, for a year, from which she was appointed night superintendent at St. Bartholomew's Hospital. From thence she joined the military nursing staff at Netley under the auspices of the National Aid Society, and in the first Egyptian campaign of 1882 was appointed superintendent sister on board the hospital ship "Carthage," receiving on her return in 1883 the decoration of the Royal Red Cross from the hands of Her Majesty, and was appointed superintendent sister to the Military Station Hospital at Fort Pitt, Chatham, in 1885-86. Miss Stewart was sent by command of the Queen to nurse the sick and wounded in the Servio-Bulgarian campaign, for which she received (with Her Majesty's permission) the medal and decoration of the Red Cross of Bulgaria in recognition of her services in connection with the National Society for Aid to Sick and Wounded in Time of War.

A NURSE'S CASE-BOOK.—We beg to announce that a case-book specially designed for nurses will shortly be issued from our office. When we published a series of articles on "Case-taking," in October, numerous enquiries were made for a suitable case-book for a nurse's pocket, and we found that such a thing did not exist. We therefore had prepared a neat little book, which will shortly be published for a shilling.

SHORT ITEMS.—Six of the St. John the Divine nurses have lately taken rooms and set up for themselves; they are doing well.—The Sisters of Mercy who have nursed at Dublin, Jervis-street Hospital, for thirty-five years have been publicly thanked by the committee for their gratuitous services.—A nurse from St. Luke's Mission, Camberwell, sails for Molokai this month to nurse the lepers.—Miss Alice Dannatt is writing a series of stories about nursing in the *Lady*.—The Duchess of Albany visited the Royal Hospital for Women and Children on December 20, and gave away toys.

A LETTER FROM THE STATES.—A nurse who emigrated to the States last autumn writes to tell us of her well-being, stating that her long silence is due to excessive work. Good news this for those nurses who think of following in her footsteps. She says: "In private nursing one has never a minute to call one's own, and here it is much worse than at home, where I had an institution and set of rules at my back. I received an offer of the post of head nurse in a new hospital that is to be opened at St. Louis, but was obliged to refuse it. I am getting on very well indeed. It reflects credit upon the English system of training that a Canadian and I stand at the head of our profession here. My last case was in what is considered the finest house in the city; it was certainly very handsome, but, like all the houses here, it lacked the cosy look of English homes. We have had lovely weather ever since we came here, and now we wonder how we ever endured the gloomy winters at home."

GRUMBLING LETTERS.—We feel inclined to say with Tennyson: "It is here, the close of the year, And with it a spiteful letter," only unluckily in our case it is many spiteful letters. First, there was a letter complaining of long hours and inconsiderate treatment of nurses at a well-known London hospital. We made enquiries, and beg to inform the writer that her statements were false, and that we discovered she had been dismissed from that hospital for insubordination. When we are trying our very best to help nurses in every way, it is very disappointing to have to investigate every letter we receive, and never to be able to believe anything stated till we have verified it personally. A grumbling, discontented woman has no business to be a nurse, and those who are dissatisfied with their calling had better take up some other. Then there is a letter which states that "We are ruined by Chinese cheap labour," or, in other words, by paying probationers. The "lady-pupil" certainly is rather overdone just now, but that is a fashion which will pass away. The statement that there are about seven nurses to one patient is ridiculous; just see the fifty and more vacancies for nurses we advertise every week. This very kindly letter of peace and goodwill concludes: "There is one comfort; if we are badly off now, the young nurses will be worse off twenty years hence." Letters written in this tone are a disgrace to the writers, and we are very sorry for those nurses who can find nothing better to do than to trump up false charges or write disparagingly about their profession and their fellow-workers.

### Notes on Opiates.

*(From the Note Book of a Nurse who attended Dr. Symes Thompson's lectures.)*

*(Continued from page l.)*

WE will now consider some of the curses that come to mankind from the use of opiates. There is no denying the fact that anæsthetics have proved dangerous to life, but nevertheless the deaths which have occurred from chloroform are very few in proportion to the number of those who have taken it. In opium, as in alcohol, the greater danger is excess, and the dangerous effect it has of weakening the self-control of its victims, so that those addicted to it lose the power of regulating the quantity taken, and each time it is indulged the greater the difficulty to resist it. From time to time we hear of cases where the evil effects of indulgence in opium are very marked, whilst many others take it with impunity. Those who have once got accustomed to it, for instance, Chinese merchants, become dispirited and listless if deprived of their opium, whereas when indulging in it they are cheerful and bright. Nor is there any great danger in opium if the person will take it in moderation and keep control over himself. In China, where rice is grown and the climate is wet and cold, and there is much ague, opium is almost universally taken. Also in the Eastern counties of England opium is largely consumed by the working classes and peasantry, who lay in a stock of it when in town on a market day, in the same way that a supply of alcohol or tobacco is laid in, and in fact, opium, takes the place of these two.

Opium is more dangerous when inhaled, as is the case in China through a pipe, whilst in England it is taken in the form of pills.

To a large extent the effect of opium is similar to that of coca, since it takes away the feeling of fatigue and does away with the close association between the person and the things around him, so that he seems to be in dreamland, and there is a want of reality in all his surroundings; bu e drawback is that in place of this absence of fatigue no real increase of strength is given, and, as opium gives a distaste for wholesome food, the waste and repair of the body is not properly balanced, with the result that the body becomes weakened.

In the body there are a series of nerves which have the control of various parts, and these controlling influences are called "inhibitory nerves," and when opium and alcohol are largely indulged in we are apt to lose these inhibitory forces, and the loss of this power may be very serious.

Among the causes which are apt to produce the opium habit are, first, heredity, and this tendency is one of the most difficult to overcome—quite as much so as the alcohol craze, and equally dangerous, since it tends to epilepsy. The next cause which induces the habit of alcohol or opium is worry or failure in business. Another cause is the desire for relief from pain, and this is by far the most excusable, for it is very natural when in great pain to desire relief from it, and opium, being the best means to secure this end, is naturally resorted to. In one case a man took opium regularly for five months for the relief of a painful tumour, during which time he certainly obtained relief, but became such a slave to the drug that he could not dispense with it. When he fully realised the depth of his bondage he decided to dispense with opium, and being very ill and nervous it was a very difficult task. One night he felt so ill that he determined he would resort to opium again, and got up for that purpose, but finding it packed up in a trunk, and the night being cold, he gave it up, and persevered in his resolution so well that he quite overcame the habit. When opium is used to alleviate pain the sense of pain following its removal is very miserable, and has been described as "feeling as if you were coming to pieces." The organism of the lower races gives way more quickly under opium than that of the superior types, and the injurious results are greater in the former than in the latter; also young people suffer more from it than adults, and its effects are more marked in those in ill health, when the powers of resistance is less than it is in good health.

Sleeplessness as a result of worn nerves frequently leads to taking chloral, bromide, and other sleeping draughts, and a few doses may be allowed, but if once it becomes a confirmed habit it is fatal. Sleeplessness should be met with rest rather than with opiates, warm baths well managed, massage, by which the blood is diverted to other parts of the body, and thus relieves the congested head, and wet rags laid on the head, or a compress on the pit of the stomach is good.

Sometimes if the refuse products are retained in the body sleeplessness is the result, since headache and pain are produced. Breathing impure air causes sleeplessness, and if opiates are given they only cause the poison to remain in the body instead of getting rid of it. Sleeplessness is, of course, to be expected in people who indulge in three or four good meals, and three or four intermediate ones in the day, and have a nap in the afternoon. More food is taken than the liver can digest, and so the peptones are carried over the body unchanged, and are quite as poisonous as the poison of ague, whereas a person leading an active life can bear and requires more food than a person leading a sedentary life. On the other hand, a person like a poor sempstress, who lives on tea and toast, and very little of that, gets her stomach into such a weak condition that it cannot digest more, and good food in small quantities is required to let the stomach regain tone. In either of these cases opium would be equally injurious. Opium is very injurious if taken in cases where the circulation in the brain is imperfect on account of heart disease, and digitalis or some other drug which would strengthen the heart would be far better. When a person works for long at a particular subject, that part of the brain so employed becomes congested, and therefore the bloodlessness of the brain necessary for sleep cannot be produced. Where there is variety of brainwork this consequence does not follow, but to grind on steadily for a long time at one subject wearies the brain and causes sleeplessness. Persons who have much anxiety and care do not always suffer from pain or sleeplessness, but from intolerable depression and a craving for some relief, which often ends in indulgence in an opiate. Opium destroys the memory, and deprives its victim of the power of accurately stating facts, and hence the proverbial untruthfulness of those who take opiates.

### "In Days of Sickness."

*(By a Patient.)*

THE line between sickness and health seems broad and strongly marked, yet, one day you may be busy with life's pleasure and life's work, the next lying on a bed of fever or of pain. The interests of outdoor life are soon wrapped in mist. For the most part the mind is absorbed with the condition of the body, and the central object of interest from the outside world is—the doctor. I like to recall in the days of health the many kindnesses I have received in sickness from my doctors and my nurses.

For days, perhaps, we have been going about with a vague sense of discomfort and unwellness; though all unwitting, already we are in the grim clutch of the spectre fever.

Then comes the day when we do not rise from bed, but send for the doctor. At once a restful feeling comes over the mind. He will know what is the matter with me, and he will soon make me well again. It is astonishing, considering how often they fail us, what confidence we have in our fellow-men; in vain has David written, "Put not your trust in princes, nor in any child of man." For in our days of sickness how we hang on the words of the physician; well may he choose them carefully, for they carry with them the call to hope or to despair. As you lie in bed, then, on this first day of inability to rise and go about your daily work, a cheery voice is heard in the passage, and the doctor enters the room with firm but quiet step. A few brief questions, a few observant glances, and delivers his opinion. The order to lie still, and do as you are bid, is received almost with gratitude, for in this strange and sudden helplessness which has befallen us the will is weakened, and bends naturally towards the strong one who can help us.

The main desire in sickness is to get well again, it is the dominant chord of all we do and all that is done for us. Little enough the sick one has to do; he has "not to question, but obey," for our doctor, kind, good, skilful, and forbearing as he is, does not like questions. And, naturally, he wants to speak the truth as much as possible. Whilst in the sick condition of mind and body of his patient, to speak the truth might often be fatal: his refuge is in "golden silence."

I have read of many heroes, I have met heroes in the flesh, but, I ask, can there be a more heroic, because self-denying, life than that of a conscientious doctor? Anyhow, the doctor is a hero to his patient. They seem to have made an agreement: we will believe in each other. I do not honestly think that a doctor can have much success with a patient who has not implicit confidence in him. For my own part I believe my doctor to be the cleverest in the United Kingdom, and I wouldn't confine myself to even that limited area. I know he makes mistakes sometimes, but that is why I believe so firmly in him. It is only the strong who can afford to make mistakes; and he is strong physically and mentally. To be strong physically is an advantage for a doctor. In your own weakness it is refreshing to look on strength. Besides, you argue (weakly, no doubt), if he can keep himself so well, why should not he make me well!

In the sore struggle with disease and death the doctor has a valuable lieutenant in the trained nurse. Often reluctantly admitted into the sick-room, she is with still greater reluctance allowed to depart. Our nearest and dearest would fain do all for us in our hours of sickness. Can any hand so well smooth the pillow, give the cup of water to the parched lips, as the hand of affection? Can any voice speak the soothing word as the voice whose tones we love so well? True, yet the unaccustomed hand grows weary of long-continued service in the sick-room, and the untrained hand makes many grievous errors; and the very love which overflows the heart tends to weaken the judgment and unnerve the mind. Surely, then, it is best for all that the trained nurse should be called to the bedside of our sick, for the nurse in some cases has a more important part to play than even the doctor.

And as you lie in your weakness and pain you are spared the extra pain of feeling that the loved one is wearing herself out in that office, which a trained nurse can fill without injury to health.

It is pleasant to watch the neat figure in its trim cap and apron flitting about the room. And when the sharp pain comes on, and your whole being is absorbed in suffering, after applying the remedies which is all that highest skill can suggest to ease the pain, she seats herself by the bedside; as you tightly grasp the ministering hand, you feel that human sympathy is a real thing. The mystery of suffering is illuminated by the power of sympathy, human, yes, and divine.

*(To be continued.)*

### Everybody's Opinion.

*[Correspondence on all subjects is invited, but we cannot in any way be responsible for the opinions expressed by our correspondents. No communications can be entertained if the name and address of the correspondent is not given, or unless one side of the paper only be written on.]*

### MONTHLY NURSE AND MIDWIFE REGISTRATION.

Nurse Harris writes: In The Hospital, Miss Heanley writes, *re* monthly nurses, "Do such nurses, as a rule, understand how to pass a catheter or give enemas? They do not." I should like to know (*a*) if the "London head midwife" is attached to any hospital; (*b*) if so, where her respect for its reputation, her sense of responsibility in her work? Against the head midwife's "no," I place a monthly nurse's "yes." Out of respect to the conscientious training, received from a London midwife, at a London lying-in hospital, will you permit me to say publicly (*a*) that enemas were given in every case before labour; (*b*) that on the third day urine, obtained per catheter by the nurses in the respective wards, were placed for analysis on the house surgeon's table.

A Monthly Nurse writes: I have read with surprise that part of Miss Heanley's letter in your last number that refers to midwives and monthly nurses. If these women as a rule do not understand how to manage the minor complications of the puerperal state, how is it that the examining physicians who know so well what that state requires grant them certificates? As a matter of fact, labour so often affects both bladder and rectum, not to mention the nervous system, that a nurse in a lying-in hospital often gets more practice with enema and catheter in two months than a probationer in a general hospital during a whole year. Again, allowing that the training might be lengthened with advantage, it must be borne in mind that one normal labour and its treatment is much the same as another, and that a certain routine is observed even in the nursing of unusual cases. I remember with gratitude the careful training I received a few years back at a London lying-in hospital celebrated for its small death-rate, and I know that both matron and head midwife made plenty of time to teach personally the duties incumbent on the monthly nurse. It was also the custom of the matron to give lectures followed by questions to her nurses, and she encouraged us to ask explanations of any particular features we had observed in our cases. As the same matron and head midwife are still at the hospital, I feel sure that one, at least, of the midwifery schools trains up its nurses in the way they should go. Then in the case of three years' practice being required of a midwife or monthly nurse before registration, would it not happen that those who were worth anything would in that time secure a sufficient connection to render them careless of that benefit? On the other side, the public might object to be practised on by the unregistered, and to obtain this experience from the training schools would be far beyond the means of most women. I confess I cannot find out the blessings of registration, and it hardly seems certain that one universal level is desirable or possible. There are good, mediocre, and bad nurses; the mediocre cannot be excluded, and a few of the bad ones may manage to pass even the ordeal of examination. A highly-trained energetic nurse, the trusted aid of the surgeon at critical operations, will be quite out of her sphere nursing a case of hemiplegia in a remote village; while a woman who is confused by the bustle of a large hospital, and kept in the background by her want of alertness and duller brain, will, by reason of her less sensitive nerves, be a far greater comfort to the irritable paralytic. And then, will a doctor or patient refuse to employ a nurse who they know to be trustworthy and capable because she is not registered? I cordially agree with Miss Heanley that the training in smaller hospitals will often stand comparison with that of the large training schools. Where there are few nurses the matron is able to take a personal interest in each, and to give valuable teaching and advice. Then the absence of students is an advantage, as the dressings fall to the nurses.

### Keeping Christmas.

King's College Hospital chapel was beautifully decorated by the sisters and nurses, and a large number were present at the service on Christmas Day. The wards were decorated, and there was the usual Christmas fare on Boxing Day. The wards were illuminated, and a short entertainment of music, singing, and recitations was given in each ward by the resident medical officers, the nursing staff, and some visitors. All passed off happily and well.

The Gateshead Children's Hospital has completed rather more than a year of its existence, and the festive season was marked by a very pleasant entertainment for its inmates. A Christmas tree laden with toys was provided, and the place, prettily decorated, had a very bright and attractive appearance. Tea was given to the children, and a number of carols were sung. The Mayor of Gateshead, the Mayoress, and many of the leading inhabitants were present. The establishment of this institution has been an up-hill work, but the results of the first fifteen months of its existence have been more than satisfactory. That the hospital was urgently needed is proved by the simple fact that since the 1st of January 71 in-patients and 811 out-patients have been treated. It is difficult to realise the amount of pain which has been removed or alleviated, and which but for this hospital could not have been properly cared for. Those who have the management of this young institution are exceedingly pleased, alike with the way in which the public are supporting it and with its internal condition. The medical officers are untiring in their attentions; and they are well supported by the active and intelligent supervision of the matron, Miss Miles.

At Bristol Children's Hospital every little inmate had a new red jacket; all the wards were nicely decorated, and there was a Christmas tree in each.

At Birmingham Queen's Hospital the decorations were grand, and mottoes of all sorts, including one "Success to our noble physicians," were put up. There was Christmas fare and a concert in one ward. Miss Roberts herself looked after the children's tree. At the General Hospital Mr. J. C. Holder gave three prizes for the three best decorated wards, and presents were distributed on December 31 from large trees. At the Children's Hospital the chaplain enacted the part of Father Christmas and distributed the toys. At the Jaffray Hospital the roast beef was carved by Mr. Jaffray himself, and Chinese lanterns were effectively used in the decorations. The medical and nursing staff are getting up some theatricals.

At Bradford the ever-generous mayor (Mr. Smith Feather) provided Christmas fare at the infirmary, the Eye Hospital, and the Children's Hospital. Gifts were also provided by other friends.

At Leicester Infirmary there was a right merry Christmas: an enchanted cave and a sham patient on a liberal diet of turkey and plum pudding being amongst the sources of amusement. Fairy lamps were extensively used.

The children's Christmas tree at the London, on Tuesday, was the occasion of a very pleasant gathering. Punch and Judy were as thrilling to the small audience as ever, and eager little hands grasped the numerous toys which Dr. Gilbart Smith and others of the medical staff distributed. There was a lovely lion that roared and opened its mouth (one of the *Truth* toys) that was highly appreciated. Tea was served in the Nurses' Home, and all the wards were open for inspection.

The theatricals at Great Ormond-street were excellent, and it was evident to the audience that the trying duties of a hospital sister do not unfit her for being a good actress. Two most pleasant evenings were spent.

### Presentation.

The nurses of the private nursing institution, Grosvenor-street, Manchester, have presented their lady superintendent, Mrs. Nicholas, with a pretty afternoon tea-set, as a further token of their sincere regard and esteem. This is the second presentation made by the nurses of the above institution to their lady superintendent.

Miss Mollett, late matron of Chelsea Infirmary, has been presented by the nursing staff with a silver plate on the occasion of her resignation.

### Death in our Ranks.

We record with regret the death of Miss Elizabeth Twining, who collected the money to build the Twining Ward of King's College Hospital. Miss Twining also partly endowed St. John's Hospital, Twickenham, and was ever to the fore in supporting medical charities. During her last illness Miss Twining was attended by Nurse Agnes, her close companion from childhood, and at the funeral Drs. Simms, Day, Leeson, Ward, Crichton, and Clark were present, also Nurses Agnes and Eva.

The death is also announced of Nurse Lucy Shaw, for five and a half years a faithful worker for the North London Nursing Association.

### Vacancies.

The following vacancies are announced:

### Nurses.

Beckett Hospital, Barnsley, £24.
Blackheath Institution.
Bournemouth Institution, £25.
Brixton Nursing Institute.
Cambridge Nurses' Home (two).
Canterbury Hospital, £25.
Central London Throat Hospital, £20.
Chelsea Infirmary, £18.
Cheltenham Institution.
Clapham Association.
Devonport Hospital (several), £25.
Eastbourne Institution.
Highbury Institution.
Hitchin Institution, £25.
Hollond Institution, Nice (several), £3 per month.
Kent Nursing Institution, £30.
Kidderminster Infirmary, £25.
Leicester Institution, £25.
Liverpool Infectious Diseases Hospital, £25.
Loughborough Infirmary.
North-Eastern Hospital, £20.
North-Western Fever Hospital, £30.
Norwich Nurses' Home.
Nurses' Home, Newcastle.
Nursing Institution, 96, 97, 98, Wimpole-street, London, W., Mr Wilson has several vacancies for thoroughly-trained nurses.
Ophthalmic Hospital, £20.
Pendlebury Hospital for Children, £20.
Rochdale Infirmary, £22.
Royal London Ophthalmic Hospital, £24.
Scarborough Institution.
Seamen's Hospital (temporary).
Sheffield Union (several).
St. Helena Home, N.W.
Windsor Infirmary (night), £25.
Workhouse Infirmary Nursing Association.

### Probationers.

Guildford County Hospital.
Hull Royal Infirmary (nine).
Newport Infirmary.
Mr. Walter's Private Hospital Manchester.
Torbay Hospital.

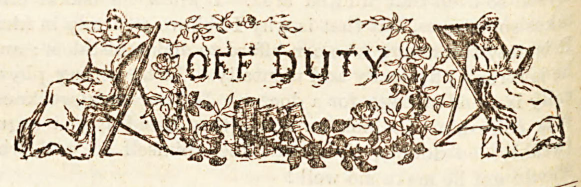

### N.B. FOURTH QUARTERLY WORD COMPETITION

### Commences Jan. 4th, 1890, ends March 29th, 1890.

Three Prizes of 15s., 10s., 5s., will be given for the largest number of words derived from the words set for dissection.

Proper names, abbreviations, foreign words, words of less than four letters, and repetitions are barred; plurals, and past and present participles of verbs, are allowed. Nuttall's *Standard* dictionary only to be used.

N.B.—Word dissections must be sent in WEEKLY, arranged alphabetically, with correct total affixed.

The word for dissection for this, the FIRST week of the quarter, being "GREETING."

### Results of Twelfth Week.

| Names. | Dec. 26th. | Totals. |
|---|---|---|
| Winifred | — | 672 |
| Adelaide | — | 646 |
| Nettie Vance | — | 757 |
| F. W. P. H. | 130 | 1673 |
| Amos | — | 596 |
| Reyot | — | 369 |
| Ellice | 107 | 1676 |
| A. S. K. | — | 275 |
| Neptune | — | 1314 |
| Ecila | 134 | 1761 |
| Tinie | — | 17 |
| Amicus | — | 946 |
| Juvenis | — | 539 |
| Jumps | 123 | 1748 |
| Sister | — | 438 |
| M. W. | 117 | 1684 |
| Esperance | 117 | 1683 |
| Sunshine | 118 | 1456 |
| Electrician | 112 | 1593 |
| H. C. | — | 562 |
| Reldas | 110 | 1653 |
| Rover | — | 776 |
| Qu'appelle | 87 | 1361 |
| Beryl | — | 188 |
| Lightowlers | 120 | 1671 |
| Jenny Wren | 90 | 1236 |
| Hercules | — | 12 |
| Night Watch | — | 346 |
| Oakenrod | — | 1170 |
| Embryo | — | 385 |
| Gypsye | — | 967 |
| Leo | — | 844 |
| Pearl | — | 299 |
| Africando | — | 171 |
| Lady Clara | — | 133 |

### Notice to Correspondents.

N.B.—All letters referring to this page which do not arrive at 139 and 140, Salisbury-court, London, E.C., by the *first post on Thursdays*, and are not addressed PRIZE EDITOR, will in future be disqualified and disregarded.

